# Genomic Analysis and Isolation of RNA Polymerase II Dependent Promoters from *Spodoptera frugiperda*

**DOI:** 10.1371/journal.pone.0132898

**Published:** 2015-08-11

**Authors:** Maren Bleckmann, Markus H.-Y. Fritz, Sabin Bhuju, Michael Jarek, Margitta Schürig, Robert Geffers, Vladimir Benes, Hüseyin Besir, Joop van den Heuvel

**Affiliations:** 1 Department of Structure and Function of Proteins, Research Group Recombinant Protein Expression, Helmholtz Centre for Infection Research, Braunschweig, Germany; 2 Department of Protein Expression and Purification Core Facility & Genomics Core Facility, EMBL Heidelberg, Germany; 3 Department of Genome Analytics, Helmholtz Centre for Infection Research, Braunschweig, Germany; Wuhan Bioengineering Institute, CHINA

## Abstract

The Baculoviral Expression Vector System (BEVS) is the most commonly used method for high expression of recombinant protein in insect cells. Nevertheless, expression of some target proteins-especially those entering the secretory pathway- provides a severe challenge for the baculovirus infected insect cells, due to the reorganisation of intracellular compounds upon viral infection. Therefore, alternative strategies for recombinant protein production in insect cells like transient plasmid-based expression or stable expression cell lines are becoming more popular. However, the major bottleneck of these systems is the lack of strong endogenous polymerase II dependent promoters, as the strong baculoviral p10 and polH promoters used in BEVS are only functional in presence of the viral transcription machinery during the late phase of infection. In this work we present a draft genome and a transcriptome analysis of Sf21 cells for the identification of the first known endogenous *Spodoptera frugiperda* promoters. Therefore, putative promoter sequences were identified and selected because of high mRNA level or in analogy to other strong promoters in other eukaryotic organism. The chosen endogenous Sf21 promoters were compared to early viral promoters for their efficiency to trigger eGFP expression using transient plasmid based transfection in a BioLector Microfermentation system. Furthermore, promoter activity was not only shown in Sf21 cells but also in Hi5 cells. The novel endogenous Sf21 promoters were ranked according to their activity and expand the small pool of available promoters for stable insect cell line development and transient plasmid expression in insect cells. The best promoter was used to improve plasmid based transient transfection in insect cells substantially.

## Introduction

The Baculoviral Expression Vector System (BEVS) is used since its development in the early 1980s [1] for high expression of recombinant protein in insect cells. It is very well established and in most cases leads to high yields of target protein [2,3]. However, as BEVS is a viral and lytic system, it impairs the cell protein synthesis machinery which might result in unfolded or incorrectly processed protein [4]. Especially proteins entering the secretory pathway often poses an extreme challenge for BEVS [5]. Consequently, alternative systems for protein production in insect cells like stable expression in cell lines [2] or plasmid based transient transfection [6] are becoming more popular. Stable expression in insect cells has already been reported for almost two decades [7], but was recently improved by establishing the Recombinase Mediated Cassette Exchange (RMCE) system in Sf9 and Hi5 cell lines [8]. Furthermore, Shen et al. [9] optimized the conditions for transient transfection using polyethyleneimine (PEI) in insect cells.

Currently, stable cell line and transient plasmid based systems are promising for protein production as for a few target proteins the yield is even higher than that achieved in BEVS [8]. However, for most proteins the yield is significantly too low. This may be due to the low copy number of the “gene of interest” in stable cell lines. In contrast, copy numbers of up to 400 plasmids per cell can be reached in case of transient plasmid based expression [2]. Hence, low promoter activity might be the bottleneck. Up to today, mostly the immediate early promoter IE1 [10] combined with the enhancer element hr5 [10–12] as well as the *Bombyx mori* silkworm actin 3 promoter [13] or the *Trichoplusia ni* pB2 promoter [14,15] have been employed for stable cell line or transient plasmid based protein expression. Unfortunately, the used early baculoviral promoters are at least 10–20 times less active than the very strong late baculoviral promoters p10 and polH [10,16]. These late promoters are dependent on several viral factors [10,16] and thus cannot be used without baculoviral infection.

In this work we attempt to solve the bottleneck of low promoter activity by testing and comparing the early viral promoters OpIE1 [17], OpIE2 [18] and hr5IE1p10 (Novagen) with endogenous *Spodoptera frugiperda* promoters. We identified a set of Sf21 promoters using both transcriptome and genome analysis of Sf21 cells. For further experiments putative upstream promoter regions were chosen based on the measured high steady state mRNA levels or by analogy to known promoters from other eukaryotic systems with high transcriptional activity.

Accordingly, the selected putative promoter regions were tested for triggering transient expression of an eGFP marker gene in Sf21 and Hi5 cells. The green fluorescence was measured in real-time using the BioLector Microfermentation system (m2p labs) in up to 48 wells in parallel, enabling high throughput and direct comparison of the efficiency of the tested promoter constructs.

In conclusion early viral and endogenous Sf21 promoters were categorized for activity in insect cells. The GAPDH and ribosomal protein L34 promoters were identified as suitable candidates for stable cell line development. The strongest promoter OpIE2 was used to improve the protein production by transient transfection. In comparison to the well-established transient expression system in HEK293-6E, plasmid based expression in Hi5 cells showed its great potential as an alternative system.

## Material and Methods

### Cell culture

BTI-Tn-5B1-4 (High5TM, Hi5, Invitrogen) cells and *Spodoptera frugiperda* (Sf21, DSMZ #ACC 119) were cultivated at 27°C in ExCell405 media (Hi5) or ExCell420 media (Sf21) respectively. Cell media were purchased from Sigma-Aldrich. Cells were maintained in exponential growth and diluted by passaging to 0.4–0.6x10^6^ cells/mL every 2 or 3 days.

### RNA isolation and sequencing

For isolation of RNA 1x10^6^ Sf21 cells were grown and harvested at 2 h, 24 h and 48 h after initial passaging of the cell culture to 0.5x10^6^ cells/mL. RNA was isolated using a RNeasyMini Kit 50 (Qiagen). The poly(A)+ mRNA fraction was isolated with oligo(dT)-magnetic beads. Libraries were prepared from mRNA using ScriptSeq- v2 RNA-Seq Library Preparation Kit (Epicentre Biotechnologies). The libraries were sequenced on a HiSeq2500 (Illumina) for 51 cycles following standard protocols. Image analysis to generate FastQ files was done with the Genome Analyser Pipeline Analysis software 1.8.2 (Illumina). Quality control and adapter clipping of the fastq sequences was done using fastq-mcf tool of ea-utils [19]. The Trinity package was used for further analysis of the mRNA transcripts [20]. A total of 30405 transcripts with a predicted open reading frame (ORF) >100 nucleotides were assembled. Transcript quantification was done with RSEM [21]. The average RSEM was 17, while 77 transcripts showed a RSEM higher than 1000. BLAST+ was used to identify 11625 protein coding regions from 30405 transcripts.

### Isolation of genomic DNA

Genomic DNA of Sf21 cells was isolated with the Genomic DNA isolation kit (Qiagen) or the AquaGenomic Kit (Multitarget Pharmaceuticals) according to the protocol of the manufacturer.

### Genome sequencing and assembly

The Sf21 DNA was sequenced by Illumina sequencing technology with two libraries: a 2x104bp paired-end library of ~280 bp inserts and a 2x94 bp mate-pair library of ~4500 bp inserts.

An initial assembly was produced with the paired-end data. SGA [22] (version 0.9.43) was used for read correction and filtering which yielded ~78.3e6 read pairs which were used as input to SOAPdenovo2 [23] (version r233) to perform contig assembly, scaffolding and gap closing.

The mate-pair data were processed with FLASH [24] (version 1.2.6) and all overlapping read pairs were discarded. The resulting ~8.7e6 pairs were used with SOAPdenovo2 for scaffolding the paired-end assembly. Both, paired-end and mate-pair data were utilized for a final gap closing step (SOAPdenovo2).

Restricting to scaffolds of minimal size 300 bp, the resulting draft assembly is composed of 51,304 scaffolds, in total 466.7 MB with an N50 of 133.8 kb (statistics computed with QUAST [25] (version 2.3)). The completeness of the assembly was assessed with CEGMA [26] (version 2.4) which detected 99.19% of ultra-conserved Core Eukaryotic Genes (CEGs) in complete copies, suggesting a very high degree of completeness. Recently, a less complete Sf21 draft genome sequence was published, comprising 358 MB sequence with an N50 of 53.8 kb and 73.79% complete CEG hits [27]. The results of the Sf21 genome assembly are shown in Table 1.

**Table 1:** The Sf21 genome assembly at a glance. Only scaffolds of minimal size 300 bp were considered. CEG hits were computed with CEGMA [26] everything else with QUAST [25].

**Number of scaffolds**	51,304
**N50 (bp)**	133,811
**Total basepairs**	466,773,710
**N's per 100 kb**	3354.37
**Largest scaffold (bp)**	1,212,604
**GC (%)**	36.22
**Complete ultra-conserved CEG hits (%)**	99.19

### Construction of plasmids containing the putative promoter region

The backbone of the plasmid pIEx/Bac-5 (Novagen) containing the eGFP gene was used to construct all expression plasmids. The hr5IE1p10 promoter of this vector was replaced either with the putative Sf21 promoter sequences or the early viral OpIE1 or OpIE2 promoter using the restriction enzyme combination *Bgl*II and *Nco*I. In case of internal *Nco*I sites within the promoter regions *Bgl*II and *BamH*I were used for cloning. The introns of the EF1 and GAPDH promoter were cloned into the same vector using the *Nco*I restriction site to generate new OpIE2 promoter-intron fusions. All required enzymes and buffers were purchased from New England Biolabs (NEB). As template for the OpIE1 and OpIE2 promoters the pIZT/V5-His vector (Invitrogen) was used (kindly provided by Paula Alves, IBET [28]). The putative Sf21 promoter sequences were amplified from genomic Sf21 DNA template by PCR using the primers shown in Table 2. Either Phusion High Fidelity DNA Polymerase (NEB) or KappaHiFi DNA Polymerase (Peqlab) was used for the PCR reaction according to the manufacturer’s protocol.

**Table 2:** Used Oligos for cloning,restriction sites are shown in bold.

Name	Sequence 3`-5`	Length [bp]
pRibo60S-for	TATA**AGATCT**GGTACAAATTAAATGTGAATTACG	34
pRibo60S-rev	TAAT**CCATGG**TTTTAATTATAAGACGTGCAAGTCGCCAACAGGC	44
pRiboL34-for	ATTA**AGATCT**GAATAAGACGGTCGACTTGGGCCAGTGTTTGTC	43
pRiboL34-rev	TAAT**CCATGG**TATGGAGCTGAAATATGAAAGATATTATAAG	41
pRiboS11-for	ATTA**AGATCT**CATGGTCTATTACCTAGCAGGCACTCCTTTACCG	44
pRiboS11-rev	ATTA**CCATGG**CTTGCTTGACAACGAAAAGAAGGTCGTGTTGC	42
pRiboL23-for	ATTA**GGATCC**GTTGTTCAGTCTCATCACCAAATGGAAAGC	40
pRiboL23-rev	TAAT**GGATCC**CGTGACAAAAAAGATGGAGGATCACTGATTATG	43
pEF1ΔI-for	ATTA**AGATCT**TCCTGGCAGATGTCGAATGTCCTTGTTTACGTC	43
pEF1ΔI-rev	TATA**CCATGG**CACGGATTACAATCCACGTGTAATATCCG	39
pEF1-for	ATTA**AGATCT**AGAAAGTATTTGGTTCCCGGAAAAG	35
pEF1-rev	TATA**CCATGG**TTTTGGTTAGTCTAGAACAA	30
pGAPDHΔI-for	ATTA**AGATCT**CGACATCAGGGTTCTGAAGACATGTTCTAAATATGAC	47
pGAPDHΔI-rev	TAAT**CCATGG**CGGAATTAATTTATGTAGTGACTGG	35
pGAPDH-for	ATTA**AGATCT**CGACAAGAGTCATGTTATACTAATATTTTC	40
pGAPDH-rev	TAAT**CCATGG**GTCTGTAATAGAAAAAAGTAAAATTATTACTAC	43
pEnolaseΔI-for	ATTA**AGATCT**CACATTATGGTATGGTCATCAAGAATAAAAGTATG	45
pEnolaseΔI-rev	TAAT**CCATGG**GAATATGATTCCGTAATTTCAGCACTCC	38
pEnolase-for	ATTA**AGATCT**CAATTACAAATTTATTAGTATACATACGGG	40
pEnolase-rev	TAAT**CCATGG**TCTGGACATGAAAACAAATAAACTCCTG	38
pActin-for	ATTA**AGATCT**GAGGGGGAAATCATCCAATGACTTCTACCG	40
pActin-rev	TAAT**CCATGG**ATTTCTAATTTCACTAAATACTGTTCTATTTC	42
pPGK-for	ATTA**AGATCT**GGGTCATCAAAGGTAAATAAATAACTGAC	39
pPGK-rev	ATAT**CCATGG**TCTGGTTAAAGTTTTTAAACTTTTTAAC	38
pHsp70-for	ATTA**AGATCT**CCTCATCCTTGTACTTCTCGGCCTC	35
pHsp70-rev	TAAT**CCATGG**CTTCGACCTCGGCGGCGGAACGTTCG	36
IntronGADH-for	ATTA**CCATGG**CGGTAAGTGCAACAATTACATG	32
IntronEF1-for	ATTA**CCATGG**GTGGTGAGTGTCAGAAGA	28

### Transfection of the insect cells with Lipofectin

Insect cells at a density of 0.3–0.6x10^6^ cells/mL were transfected with the respective expression plasmid using Lipofectin Transfection Reagent (Invitrogen, Carlsberg, CA). A DNA concentration of 2 μg per 1x10^6^ cells was used at a ratio of Lipofectin: DNA of 2:1. DNA and Lipofectin were incubated with medium for precomplexing in 2.5% of the final volume. The mixture was added to the cells after 30–60 min of incubation at RT.

### Insect cell cultivation using the BioLector

The BioLectorBasic Microfermentation system (m2p labs) enables a direct comparison of optical cell density and green fluorescence of up to 48 different cultures. Thus, allowing a direct comparison between the efficiency of eGFP expression for the different putative promoter regions. Gain levels of the sensors were selected for measuring in a linear range. The optical density correlated directly to the cell concentration up to 3x10^6^ cells/mL. The transfected cells were cultivated in a volume of 2 mL at 700 rpm, 27°C and 85% humidity. The measured eGFP intensity was blanked against a culture transfected with a control plasmid solely expressing mCherry.

### Transient transfection and cultivation of HEK293-6E cells

Cultivation of HEK293-6E [29] was performed in F17 media supplemented with 25 mg/L G418, 1 g/L Pluronic F86 and 7.5 mM L-glutamine in an Infors Minitron orbital shaker at 100 rpm at 37°C in a 5% CO_2_ atmosphere. For transfection 2 μg pFlpBtM-II-eGFP [30] plasmid DNA per 1x10^6^ cells were incubated in 200 μL F17 media according to the standard transfection conditions as described by Bollin et al.[31]. The HEK293-6E cells were seeded in a total volume of 1.8 mL at a concentration of 0.6x10^6^ cells/mL and afterwards the 200 μL transfection mix was added. The cells were cultivated in a 48 well plate in the BioLector at 37°C, 700 rpm, 85% humidity and 5% CO_2_.

### Flow cytometry

The Guava flow cytometer (Merck Millipore) was used to determine the transfection efficiency and to confirm the fluorescence data obtained by the BioLector. Cells were diluted 1:10 in PBS and dead cells were stained red with Propidiumiodid (0.05 mg/mL). Untransfected cells were used as negative control for transfection efficiency.

## Results

### Transcriptome analysis and selection of putative Sf21 promoter regions

The transcriptome of exponentially growing Sf21 cells was analysed for early, mid and late log phase. Transcripts were ranked according to their abundance using the RSEM value. Open reading frames were analysed by BLAST+ comparison, identifying more than 11000 different proteins. Selected mRNA sequences were aligned to the draft genome of Sf21. The most promising promoters regions were selected based on expression level and orthologous comparison. Genes with a high steady state mRNA level with a RSEM of more than 3000 compared to the average value of 17 might indicate a strong promoter. However, a high expression level could also be caused by other factors, like high mRNA stability. Altogether four putative promotors were tested, which all belong to the class of the high abundant ribosomal protein mRNA. In a second approach, Sf21 promoters regions were chosen, based on the existence of strong promoters for orthologous proteins from other eukaryotic organisms These include Elongation Factor 1 (EF1) [32], Enolase [33], Heatshock protein 70 [34], Glyceraldehyde-3-phosphate dehydrogenase (GAPDH) [35] and Actin [36]. Indeed, the Enolase (RSEM 1731) and the EF1 promoter (RSEM 3076) showed a transcript level above the average RSEM. Additionally to the two groups of endogenous Sf21 promoters, three early viral promoters were tested for expression in the absence of baculoviral infection, respectively the hr5IE1p10 (AcMNPV, derived from pIEX/Bac-5, Novagen), the OpIE1 and the OpIE2 promoter (OpMNPV, derived from pIZT/V5-His, Invitrogen).

As currently nothing is described about promoter structures in *Spodoptera frugiperda*, the length of the endogenous Sf21 putative promoters including enhancing regions had to be estimated. The length of the promoter region may range up to thousands of basepairs [37]. As all immediate early viral promoters functional in Sf21 cells have a size of 200 bp up to 600 bp, in this work sequences of approximately 1000 bp upstream of the start codon were tested for promoter activity,

Some of the selected endogenous Sf21 promoter regions contain a potential intron in the leader sequence of the mRNA. To test the influence of these introns on the promoter strength, different constructs with and without an intron (ΔI) were generated. In case of the GAPDH promoter the intron was around 1000 bp long, therefore the tested promoter region was increased to a length of 1800 bp.

**Fig 1:**
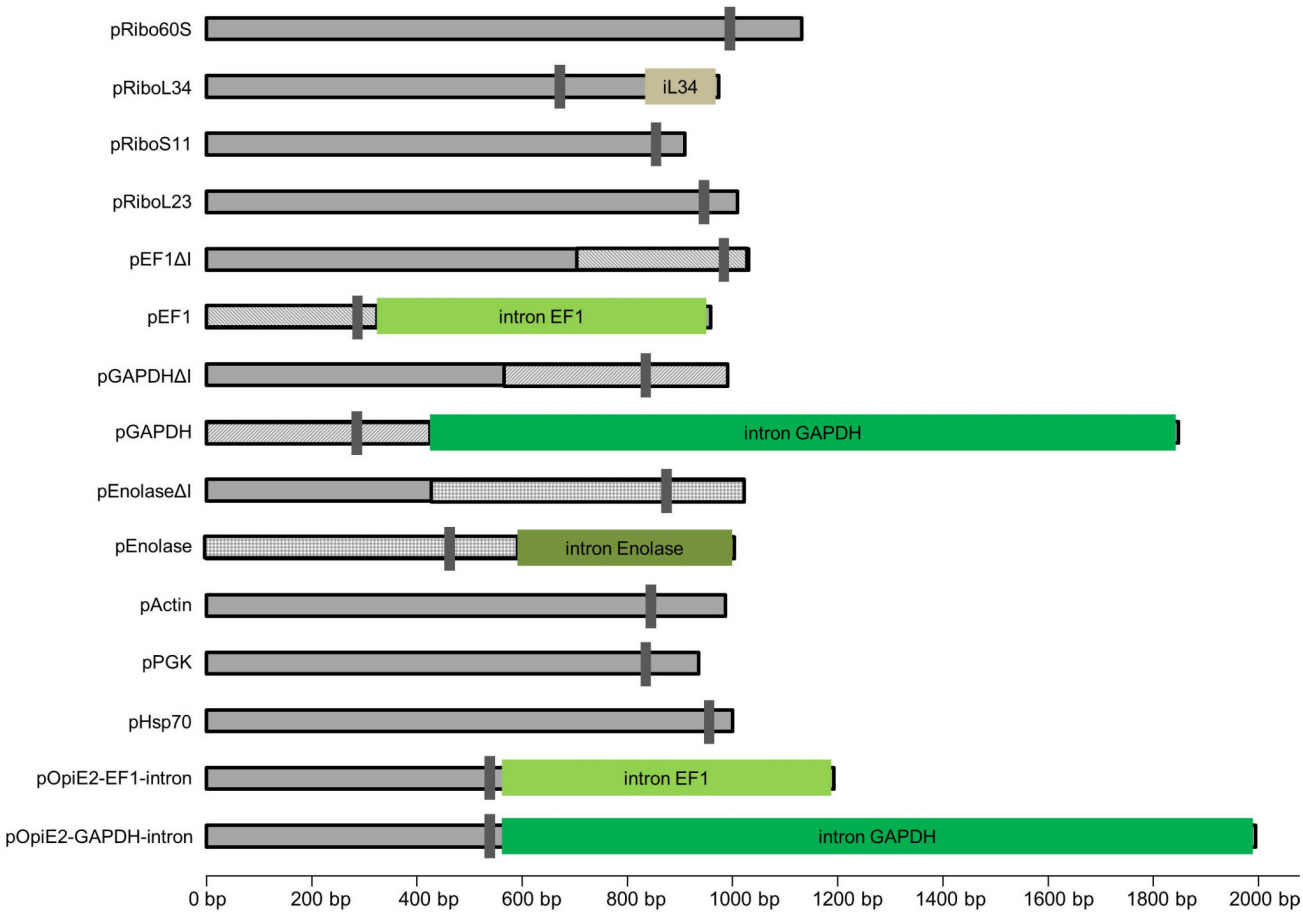
Schematic overview of the selected putative SF21 promoter regions. Shown are the predicted length, transcription start site and possible introns in leader sequence. Hatched regions highlight identical sequences in constructs with and without an intron.

All selected putative promoter regions are shown in Fig 1 and Table 3.

**Table 3:** Putative Sf21 promoter regions: The upstream regions were either chosen because of high mRNA levels or high expression of corresponding genes in other eukaryotic systems.

Group	Name	Protein	RSEM	Predicted Length
Highest transcript-level	pRibo60S	60S Acidic ribosomal protein P1	7556	1130 bp
	pRiboL34	Ribosomal protein L34	6388	972 bp
	pRiboS11	40S Ribosomal protein S11	5218	908 bp
	pRiboL23	Ribosomal protein L23A	3376	1008 bp

Strong analogous promoters	pEF1ΔI	Elongation factor 1-α	3076	1029 bp
	pEF1	Elongation factor 1-α	3076	957 bp
	pGAPDHΔI	Glyceraldehyde-3-phosphate dehydrogenase	396	989 bp
	pGAPDH	Glyceraldehyde-3-phosphate dehydrogenase	396	1844 bp
	pEnolaseΔI	Enolase	1731	1020 bp
	pEnolase	Enolase	1731	1002 bp
	pActin	Actin	58	985 bp
	pPGK	Phosphoglycerate kinase	35	934 bp
	pHsp70	Heat shock protein 70 A1	19	1034 bp

Early viral promoters	hr5IE1p10	Combination of enhancer hr5, early viral promoter IE1 and the very late promoter p10 (AcMNPV)		1197 bp
	OpIE1	Early viral promoter (OpMNPV)		292 bp
	OpIE2	Early viral promoter (OpMNPV)		553 bp

Combination with OpIE2	OpIE2-IntronEF1	Viral protein + EF1-intron		1191 bp
	OpIE2-IntronGADH	Viral protein +GADH-intron		1981 bp

### Cultivation and eGFP analysis in the BioLector

All promoter constructs were initially tested in Sf21 and later in Hi5 cells by transient transfection. The growth of the cells was followed by optical density and the triggered eGFP expression was measured online by the green fluorescence in the BioLector for up to 48 cultures in parallel.

The transfection efficiency was determined using flow cytometric analysis. Transfection efficiency was similar within one experiment in different cultures, whereas between repeated experiments it showed slight differences depending on cell cycle and passage number of the cells. To be able to compare the individual BioLector runs, a corrected eGFP yield was calculated taking into account both the transfection efficiency as well as the optical density. Briefly, the increase in fluorescence relative to the background was corrected for the fraction of transfected cells and related to the cell density according to the following equation.

$$Yield=\frac{BlankedGFP\;Gain50\;\times \;1000}{OD\;Gain30\;\times \;Transfection\;Efficiency\;}$$

**Fig 2:**
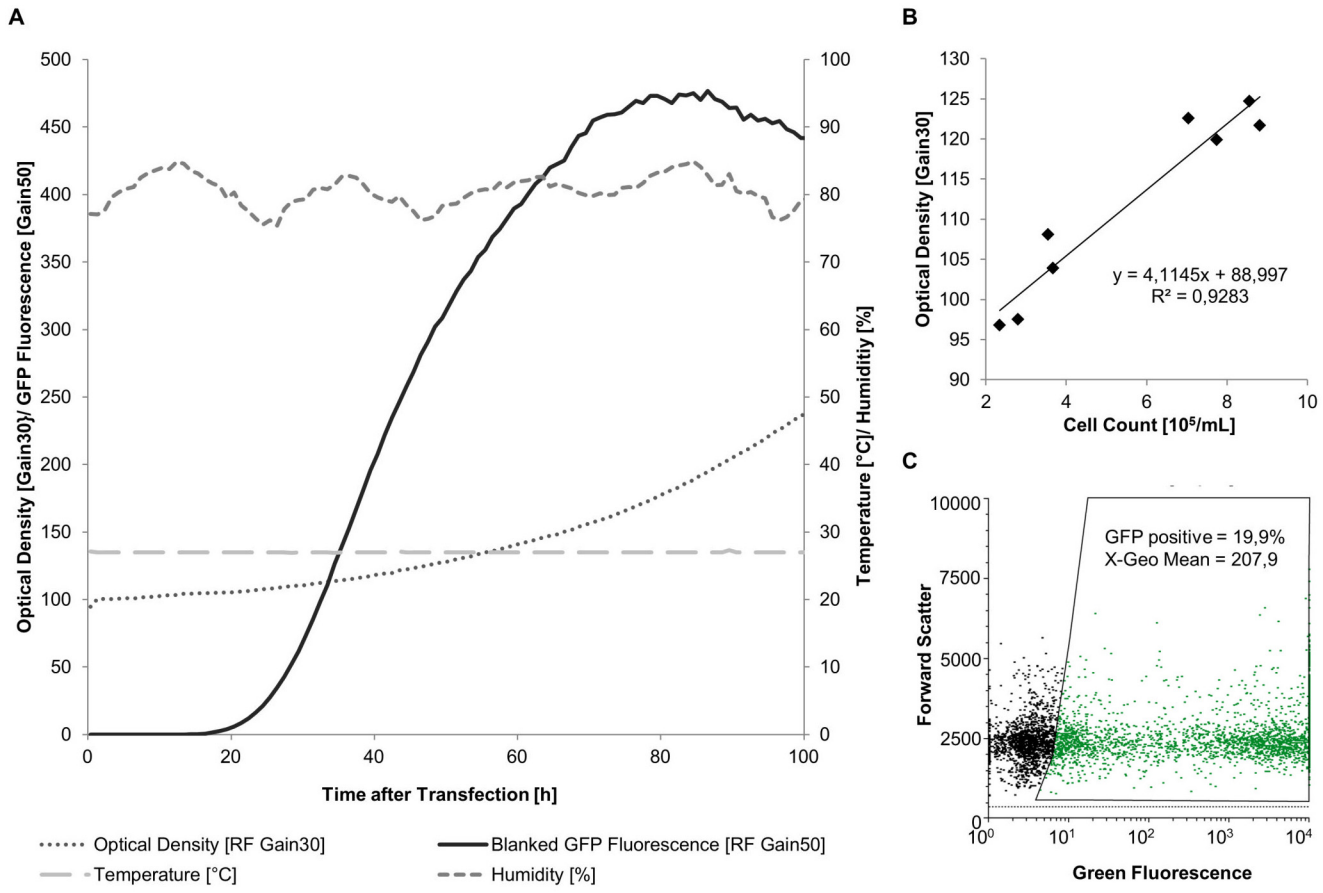
Cultivation and evaluation of eGFP expression using the BioLector system. a) Cultivation in the BioLector showing stable temperature at 27°C, humidity at around 85%, growing cell density and increasing eGFP fluorescence for Hi5 cells transfected with the OpIE2-eGFP plasmid b) Correlation between cell density measured in BioLector and cell number measured in Guava c) Transfection efficiency measured in the Guava 52 h after transfection. A transfection efficiency of 20% was reached in this experiment.

The cultivation conditions and measured parameters in the BioLector for the example of Hi5 cells transfected with the pOpIE2-eGFP plasmid are shown in Fig 2. Temperature (27°C) and humidity (~85%) was maintained constant over the whole cultivation process. The biomass of the cultivated insect cells increased over time, showing a lag phase in the first 40 h of cultivation but then growing exponentially. The counted cell numbers correlate to this measured optical density. The expression of eGFP was only detectable after 20 h, strongly increased until 72 h and reached a peak after 80 h.

### Determination of the promoter activity in Sf21 cells

**Fig 3:**
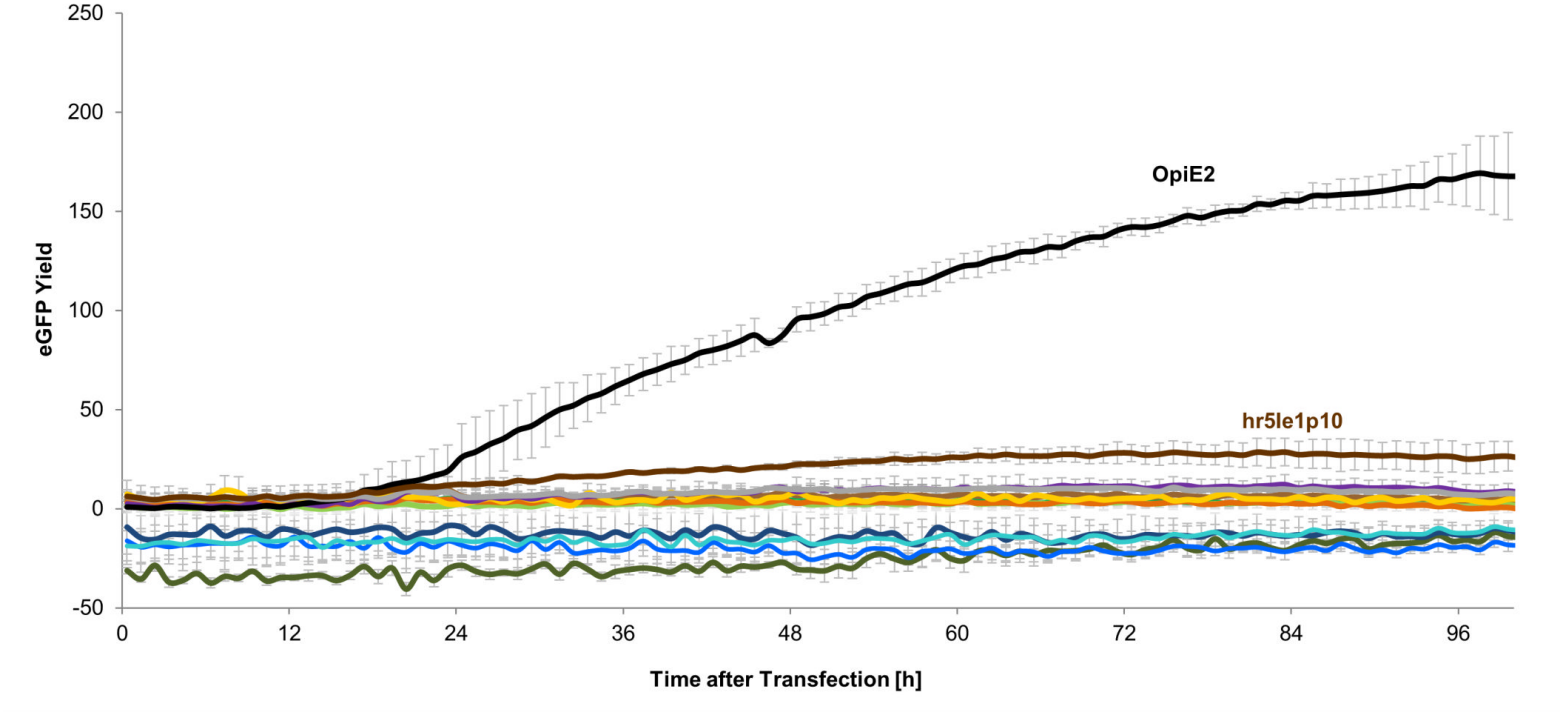
Promoter activity in Sf21 cells over time measured in the BioLector. Only the baculoviral promoters OpIE2 (black) and hr5IE1p10 (brown) showed detectable eGFP expression.

All selected promoters were transiently tested in Sf21 cells (Fig 3). None of the endogenous Sf21 promoters showed significant eGFP expression. Merely for the hr5IE1p10 and the OpIE2 promoter expression could be observed, whereby the OpIE2 promoter exceeded the hr5IE1p10 activity approximately 6 times.

### Analysis of the activity of endogenous Sf21 promoters in Hi5 cells

**Fig 4:**
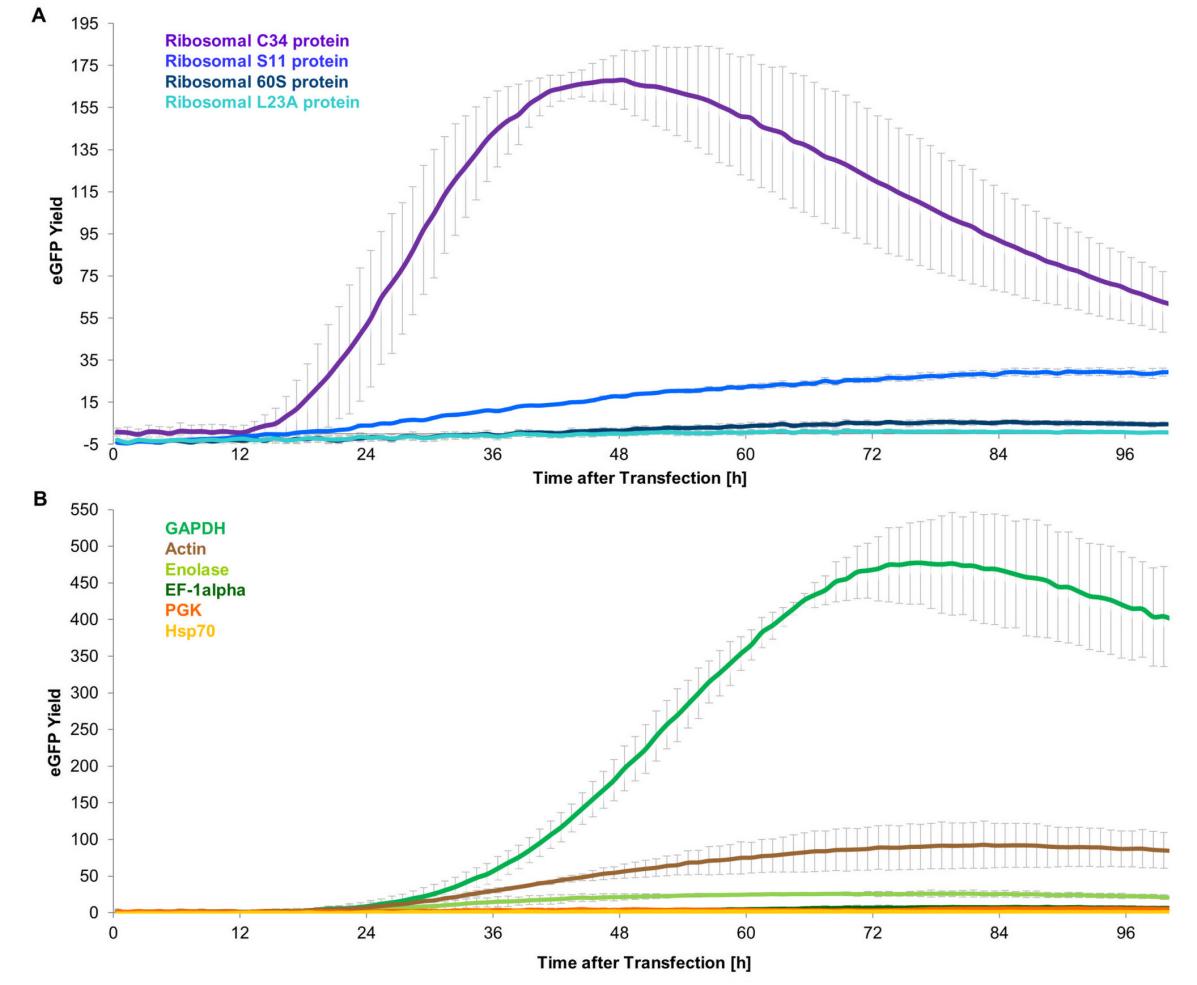
Measured activity of the endogenous Sf21 promoters in Hi5 cells in the BioLector. (A) Comparison of all endogenous Sf21 promoters with high steady state transcript level. (B) Comparison of all endogenous Sf21 promoters with highly active analogues in other eukaryotic systems.

The expression in Hi5 cells for BEVS is shown to be substantial higher than in Sf21 cells [38]. Notwithstanding that Hi5 cells (*Trichoplusia ni*) are fairly related to Sf21 cells (*Spodoptera frugiperda*), tests with the OpIE2 promoter showed 20 times increased transient expression in Hi5 compared to Sf21 (Figs 3 and 4). Therefore the endogenous Sf21 promoters were also tested in Hi5 cells. Indeed a much higher eGFP expression for the endogenous promoter constructs was observed in Hi5 compared to Sf21 (Fig 4).

Out of the high transcript level promoters, the ribosomal L34 protein promoter showed the highest activity. Furthermore, eGFP expression could also be detected for the ribosomal S11 protein promoter. The GAPDH promoter was the most active promoter out of the group of strong analogous promoters, followed by the Actin and the Enolase promoter. For the Hsp70, the PGK and the EF1 promoter only background activity could be observed.

Of all endogenous Sf21 promoters GAPDH was the strongest promoter with a max. eGFP yield of 477 and exceeded the ribosomal L34 promoter round about 2,8 times (max. eGFP yield of 168). No other endogenous Sf21 promoter exceeded a max. eGFP yield of 100.

A closer look at the eGFP expression over time revealed differences in the onset and course of expression for the different promoters (Fig 4). The GAPDH promoter derived expression for example started about 12 h later than the ribosomal L34 protein promoter. Additionally, the ribosomal L34 protein promoter reached a maximal expression after ~48 h whereas the maximum for GAPDH was hit after ~74 h after transfection. Apparently, the promoters are differentially regulated.

### Influence of the intron in the mRNA leader sequence on eGFP expression

**Fig 5:**
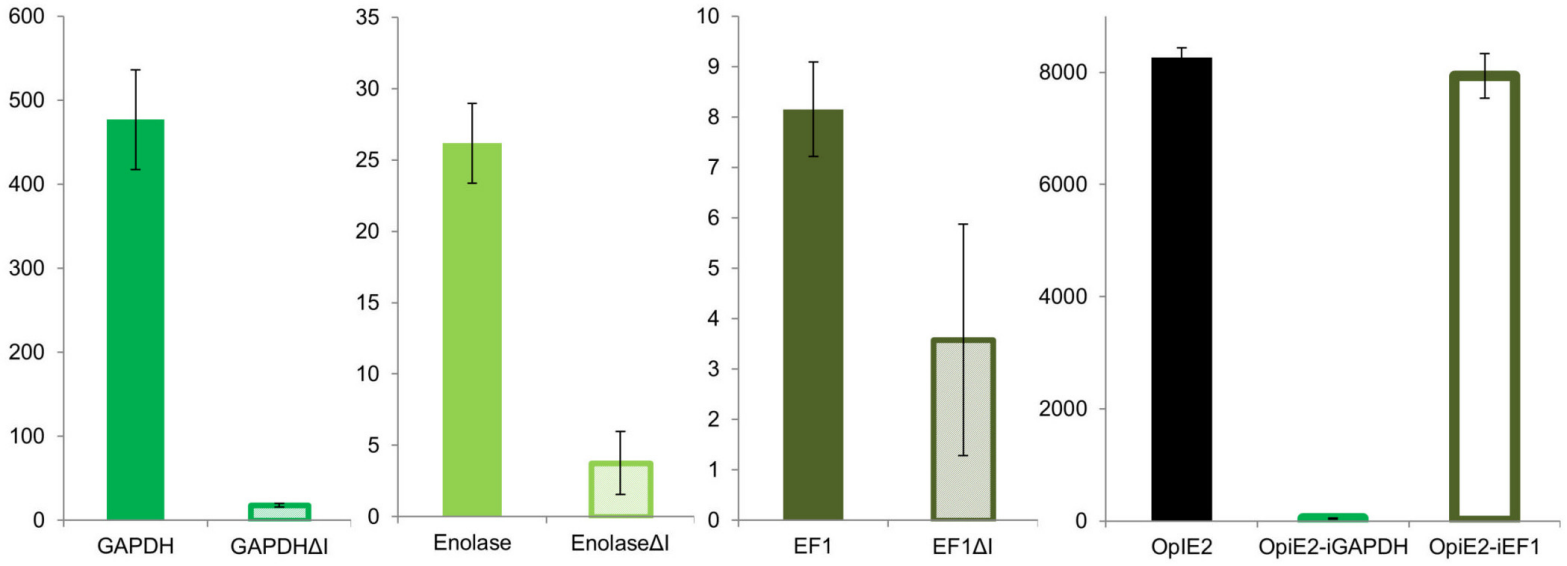
Influence of the intron on the respective promoter as well as on the OpIE2 promoter in Hi5. Deletions of the intron sequence for all three promoters led to a decrease in activity. Fusion of the GAPDH or the EF1 intron downstream to the OpIE2 promoter did not enhance the activity. On the contrary the fusion to GAPDH intron decreased eGFP expression completely.

A detailed analysis of the structure of the selected Sf21 promoters revealed that four of these ten promoters contain an intron in the mRNA leader sequences. Intriguingly the two best promoters, the GAPDH as well as the ribosomal L34 protein promoter comprise an intron. The influence of the presence of the intron for the expression in three of these promoters was tested. Indeed the introns appear to have a positive influence on the expression level. Constructs lacking the intron sequence showed a dramatically reduced level of eGFP expression (Fig 5). To test a positive effect on expression of the intron sequences, the GAPDH and EF1 introns were fused downstream of the OpIE2 promoter. Neither, the GAPDH intron nor the EF1 intron enhanced the activity of the strong OpIE2 promoter. On the contrary, for the OpIE2-GAPDH intron combination the promoter activity was completely disabled (Fig 5).

### Comparison of the endogenous Sf21 promoters and the early viral promoters in Hi5 cells

**Fig 6:**
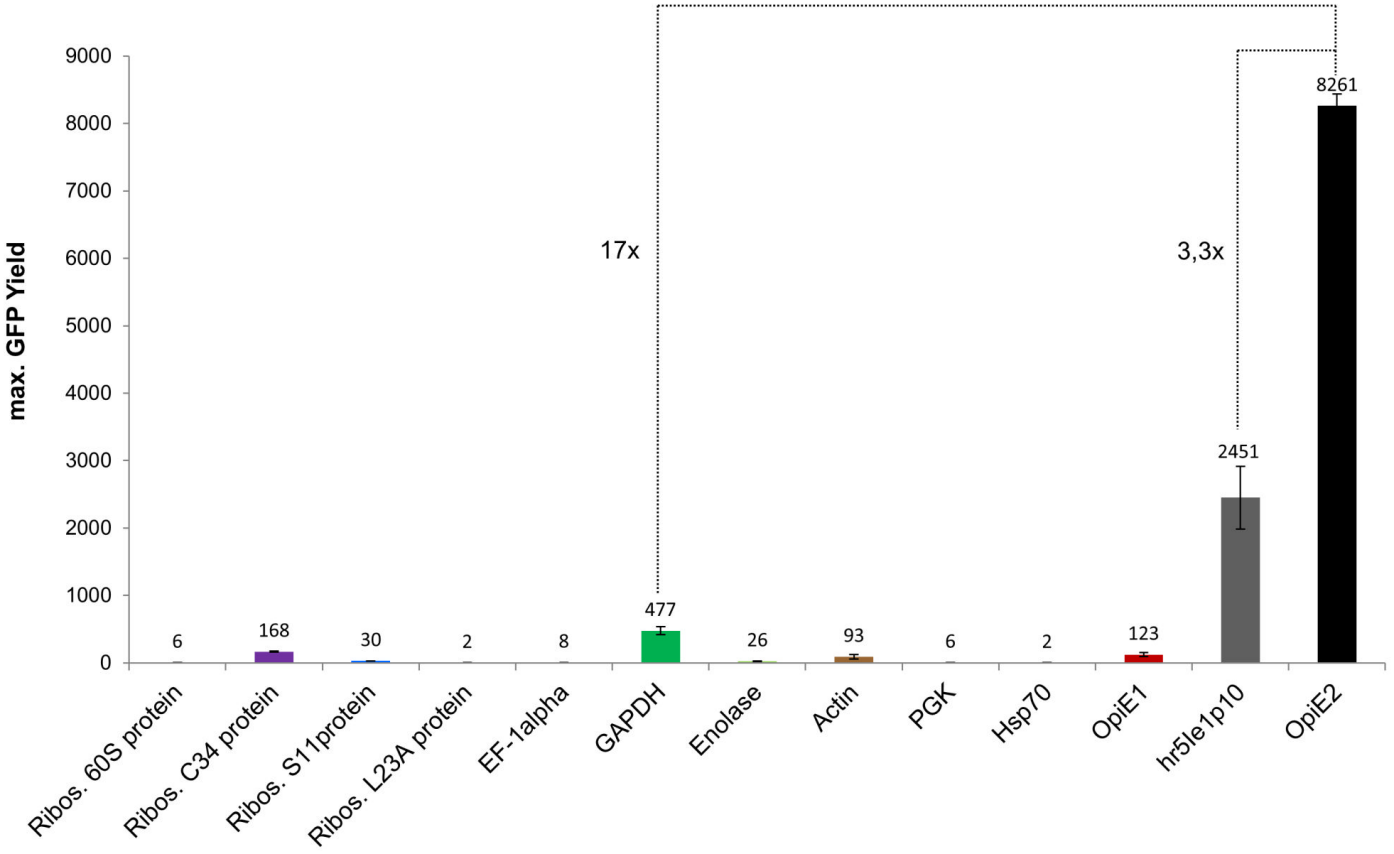
Maximum eGFP yield for all endogenous Sf21 promoters and early viral promoters in Hi5 cells.

The maximum eGFP expression of all endogenous Sf21 promoters and the early viral promoters is depicted in Fig 6. Like in Sf21 cells the OpIE2 promoter again showed the highest activity, followed by the hr5IE1p10 promoter combination which was ~3.3 times weaker. In comparison with the early viral promoter activity the GAPDH as strongest endogenous Sf21 promoter was ~17 times less active than the OpIE2 promoter. However, in Hi5 cells the expression of GAPDH promoter was ~4 times higher than the expression achieved with the early viral OpIE1 promoter. Additionally, the ribosomal L34 protein promoter showed expression in the range of the OpIE1 promoter. Thus both GAPDH and the ribosomal protein L34 promoters present genuine Sf21 promoters which can be used in insect cells for example to trigger expression of selection markers in stable cell lines.

### Comparison of the Hi5 and HEK293-6E transient expression systems

**Fig 7:**
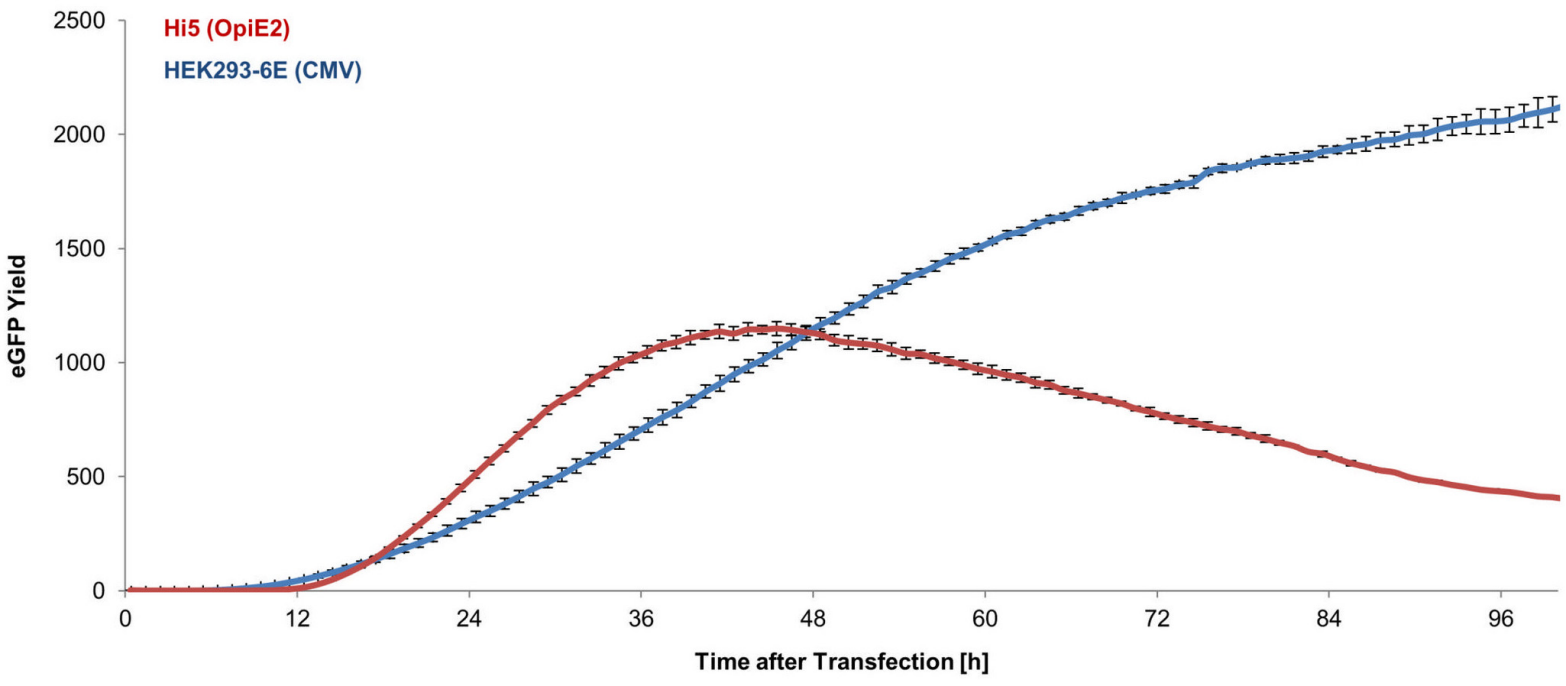
Comparison of transient plasmid based expression in Hi5 cells with the OpIE2 promoter and HEK293-6E cells with the CMV promoter.

The performance of the OpIE2 promoter as an early viral promoter was promising high in both Sf21 and especially Hi5 cells. To outpoint the high level of expression, we compared the expression in the transient Hi5 cell system using the OpIE2 promoter to the HEK293-6E system [39]. Both expression systems were compared in the BioLector based on their eGFP expression. The GFP Gain had to be lowered to 40 to stay in the linear range of the BioLector. Again a corrected eGFP yield was calculated to make sure differences were not due to biomass or transfection efficiency (For Hi5 cells = 59%, for HEK293-6E = 51%). The course of transient expression for Hi5 differed form the one for HEK293-6E, due to the presence of the EBNA system in HEK293-6E cells (Fig 7). Hence, expression in Hi5 did not show the long-term stability of the plasmid and therefore decreased after 48 h. However, 50% of the maximum yield in HEK293-6E cells could be reached using the Hi5 cell system presented in this work

## Discussion

To improve the tools for stable insect cell line engineering, substantial knowledge of the genomic structure as well as the transcriptome of *Spodoptera frugiperda* is essential. Here we analysed the transcriptome at different stages of growth and determined the genome sequence to a coverage of 99% of ultra-conserved core eukaryotic genes. This is the most complete sequencing result of the Sf21 genome up to now. These data can be used for many biotechnological applications like targeted genome manipulation or identification of site specific integration loci.

We used the transcriptome and genome data to identify a set of putative Sf21 promoter regions and analysed the activity by transient plasmid-based transfection in insect cells. As a result we were able to expand the pool of available promoters for stable insect cell line development with the new Sf21 GAPDH and ribosomal protein L34 promoters. These are suitable to activate selection marker expression in stable cell line development, because their activity is in the range of the OpIE1 promoter activity [8]. Furthermore, the Hsp70 promoter was tested without any kind of induction, which might be the reason for its rather low basal activity. Noticeable promoters with highest activity were not necessarily those with a high RSEM level, as the RSEM value did not directly correlate to eGFP expression. This could be caused by differences in mRNA turnover rates, where some mRNAs might be more stable than the other and thus having a high RSEM level but still are still depending on a weak promoter. Therefore, more putative Sf21 promoter regions have to be tested, to identify promoters with even stronger activity than those presented in this paper. Fortunately, the early viral OpIE2 promoter showed a substantial high expression activity in our analysis, much higher than published for an early viral promoter or hybrid viral promoter combination before.

To get a better insight in the promoter structure further characterization of the promoter architecture is inevitable. Classification of enhancing or inhibiting regions is necessary but difficult as so far no binding sites for transcriptional factors are identified in *Spodoptera frugiperda*. Hence, classical deletion studies to identify the key sequences for transcriptional regulation elements remain presumably the best method for promoter characterization. These sequences could also include regions far more upstream than 1000 bp as enhancers can be located in a distance of up to 100 kbp of promoter sequences in eukaryotes [37]. Furthermore, in this work introns in the 5’region of the mRNA obviously play an important role in promoter activity, as activity was dramatically decreased when the corresponding intron was deleted. The combination of these introns with the early viral promoter OpIE2 did not lead to an increase in activity. Therefore, unknown interactions between the intron and corresponding promoter sequences seem to occur which were not valid for the interaction with the OpIE2 promoter.

Interestingly, Hi5 cells outperformed Sf21 cells in plasmid based transient expression of eGFP up to 20 times, in the case of the OpIE2 promoter. Even with host-endogenous Sf21 promoter regions Hi5 cells clearly exhibit higher yields. The same phenomenon was observed but not to this extend in BEVS expression [38]. This might be caused by differences in metabolic activity of the different cell lines [40].

Comparing the yield of our plasmid based transient transfection to BEVS, the BEVS reach higher expression levels but is more time consuming and costly. However, in the presented plasmid based system substantial expression was shown. Therefore this system is an economical alternative for fast screening of constructs and it will not be necessary to use the so called “Transactivation” method for screening as published by Radner et al. [41].

In relation to the very well established and often used plasmid based expression system in HEK293-6E cells, transient expression in Hi5 using the OpIE2 was encouraging high. Despite different expression profiles due to the lack of the EBNA system in Hi5 a level of 50% of expression of intracellular eGFP compared to the HEK293-6E system could be reached. Taking media and licence cost into account, Hi5 cells show a great potential as cheaper and efficient alternative to the transient HEK293-6E cell system.

In conclusion this study showed the potential of plasmid based expression in Hi5 insect cells in combination with the right choice of promoter. Additionally, two new Sf21 promoters were identified, which can be applied for stable insect cell line engineering.
